# An inertial mechanism behind dynamic station holding by fish swinging in a vortex street

**DOI:** 10.1038/s41598-022-16181-8

**Published:** 2022-07-25

**Authors:** Sam Tucker Harvey, Valentine Muhawenimana, Stephanie Müller, Catherine A. M. E. Wilson, Petr Denissenko

**Affiliations:** 1 Department of Engineering Science, University of Oxford, Oxford, OX1 3PJ UK; 2 School of Engineering, Cardiff University, Cardiff, CF24 3AA UK; 3 School of Engineering, University of Warwick, Coventry, CV4 7AL UK

**Keywords:** Fluid dynamics, Bioenergetics, Ichthyology

## Abstract

Many aquatic and aerial animal species are known to utilise their surrounding flow field and/or the induced flow field of a neighbour to reduce their physical exertion, however, the mechanism by which such benefits are obtained has remained elusive. In this work, we investigate the swimming dynamics of rainbow trout in the wake of a thrust-producing oscillating hydrofoil. Despite the higher flow velocities in the inner region of the vortex street, some fish maintain position in this region, while exhibiting an altered swimming gait. Estimates of energy expenditure indicate a reduction in the propulsive cost when compared to regular swimming. By examining the accelerations of the fish, an explanation of the mechanism by which energy is harvested from the vortices is proposed. Similar to dynamic soaring employed by albatross, the mechanism can be linked to the non-equilibrium hydrodynamic forces produced when fish encounter the cross-flow velocity generated by the vortex street.

## Introduction

Aquatic and aerial animal species are well known to utilise their surrounding flow field and/or the induced flow field of a neighbour to reduce their physical exertion, enabling them to travel over longer distances. Examples include hydrodynamic drafting observed in mother–calf dolphin pairs^1^, drafting used by ducklings and spiny lobsters moving in formation^2,3^, ibis maximising upwash through v-flock flight formation^4^, and cross-flow dynamic soaring employed by albatross^5^. Such precise, complex flight and swimming dynamics require high-skilled manoeuvring and hydro- and aerodynamic mechanisms to benefit their motion, which are currently only partially understood.

The perfectly synchronised movement of fish swimming together in an organised pattern, known as shoaling or schooling, is exhibited by many fish species, yet surprisingly limited comprehensive hydrodynamic explanation for the benefits of collective swimming exists. Broad explanations have been offered based on the follower fish capturing energy from vortices generated from the leading fish to reduce energy expenditure^6,7^. One of the earliest explanations postulated was that the follower fish could gain thrust derived from the induced flow-stream between two parallel reverse Kármán vortex streets generated from a pair of leading fish^7^, although this theory only works for a specific relative positioning of three fish. Recent studies have shown that leader swimmers also derive energy benefits from followers at close distances^8–10^. However, it is well known that shoaling fish do not adopt a fixed distance swimming formation and that the nearest neighbour distance is continuously changing^8,11,12^. Additionally, computational studies and modern machine learning methods have revealed relative positioning and kinematics that allow both the leader and follower in a fish pair to obtain an energy benefit. By combining high fidelity flow simulation with reinforcement learning algorithms, Verma et al.^13^ demonstrated that an off-centre following position was energetically advantageous, rather than in-line swimming.

The case of fish swimming behind wake-forming obstacles such as vertical cylinders has been studied extensively, where fish alter their locomotion by synchronising their swimming gait with the unsteady Von Kármán vortex street^14–17^. The term “Kármán gaiting” was coined for fish tuning their body wavelength and lateral translation to match the Von Kármán vortex shedding frequency^18–20^. Although Kármán gaiting has been shown to be energetically advantageous for fish by reducing their energy costs and enhancing their swimming efficiency^14,17,21^, with the mean velocity in the wake of objects generating the vortex street being lower than in the free stream, decoupling benefits of Kármán gaiting and staying in areas of calmer flow has not been feasible. While flow structures in Kármán gaiting appear similar to those in collective swimming, a key distinction can be made in terms of characteristic shedding frequencies. Collective swimming typically occurs close to the natural tailbeat frequency for a particular species^22^, however, in the case of Kármán gaiting, an additional oscillatory component appears at a frequency offset from that of regular swimming.

A number of studies have focused on fish swimming in pairs to gain further understanding of the hydrodynamic incentives of collective swimming. The oscillating motion of a leader fish swimming in carangiform mode, creates a thrust generating reverse Kármán vortex street in its wake^23,24^, and experiments have replicated this vortex system using robot fish and oscillating aerofoils to control the wake dynamics while using real fish in the wake as the follower fish^22,25,26^. Such experiments have highlighted two independent kinematic behaviours, one, that the follower fish adjusts its tail beat frequency to match that of the leader fish, and two, the follower’s station holding depends on the relative spatial positioning^25,27^. The recent study by Li et al.^22^ has advanced this understanding by showing that the tail beat/vortex frequency matching of the follower fish to that of the leader fish is maintained at a precise phase difference, which is a function of the relative spatial positioning.

In this paper, we provide a linked hydrodynamic and kinematic explanation of the use of the periodic cross-flow generated in a thrust generating Karman vortex street by juvenile rainbow trout (*Oncorhynchus mykiss*). By superimposing flow visualisation and velocity measurements with fish tracking data, new hydrodynamic insight is obtained, highlighting inertia as the main mechanism for dynamic station holding.

## Results

**Figure 1:**
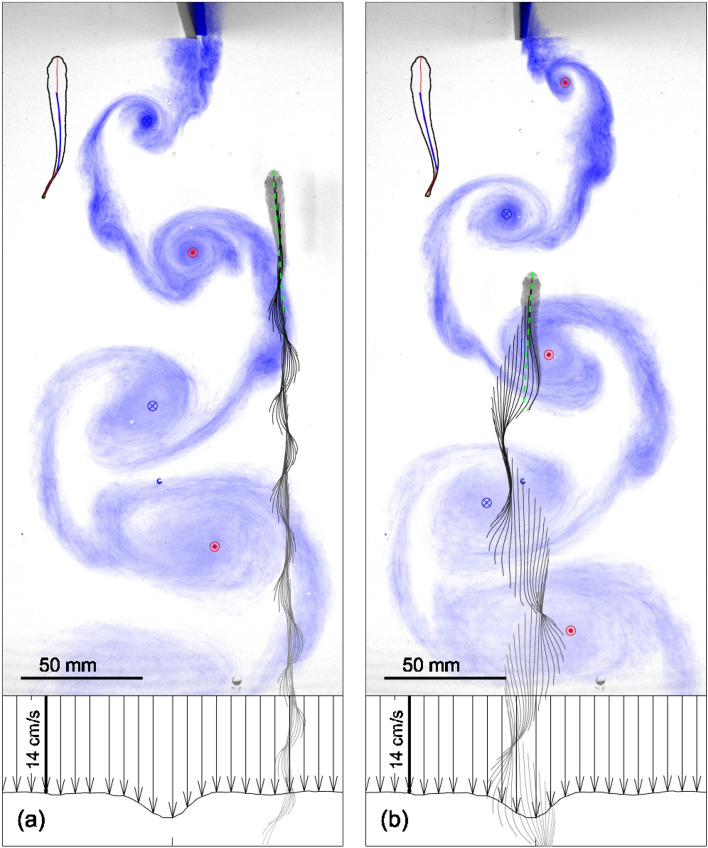
Fish exhibit two distinct swimming gaits: (**a**) regular swimming outside the wake in the uniform flow field and (**b**) swinging between vortices. Sequential positions of the fish centreline advected downstream with the mean flow velocity are imposed on the ink visualisation of the reverse Kármán street, obtained in separate experimental series. Profiles of the mean streamwise flow velocity are shown below the images. In the top left corner, the contour of the fish body is shown with the part of the centreline associated with muscle marked blue.

Experiments were performed with juvenile rainbow trout swimming in the thrust generating reverse-von Kármán vortex street formed by a hydrofoil oscillating in a uniform flow of 0.14 m/s. The frequency of the hydrofoil was varied from 1.2 to 2.5 Hz. Most fish spent the majority of the test time swimming regularly outside the wake and probing the flow field by crossing the wake and choosing the optimal location for station holding. Based on this observation, two distinct swimming regimes were identified: regular swimming outside the vortex street and swinging across vortices within the inner region of the wake, as shown in Fig. 1a,b respectively, disregarding any irregular swimming motions such as bursts, drifts, turns, and station holding outside the wake. Of the 34 individual fish observed during the testing, three exhibited a sustained altered gait (i.e., swinging) whilst interacting with the vortex street generated by the hydrofoil. The profiles of mean flow velocity displayed at the bottom of the plots show that, whilst swimming in the Kármán street, the fish encounters flow velocities of up to 20% higher than in the unperturbed flow. As rainbow trout have typically been observed to navigate to regions of lower mean flow velocity, where their energy expenditure is minimised^28^, this suggests that fish are able to reduce the propulsive cost in spite of the higher velocity in the wake region by exploiting the flow structure of the vortices. The kinematics of the altered gait bares resemblance to Kármán gaiting, first reported in^15^, which is characterised by a large cross-stream displacement of the fish in phase with the vortices.

When neither swimming regularly nor swinging between vortices, the fish have been observed to probe the wake whilst attempting to synchronise their motion with the flow structures. Occasionally, when failing to synchronise with vortices, the fish perform a sudden manoeuvre, leaving the vortex street. This behaviour can be observed when reviewing supporting videos.

**Figure 2:**
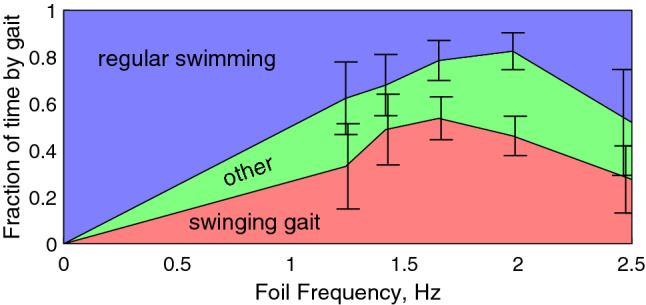
Proportion of time spent in different swimming modes, averaged between the three individual fish which exhibit the swinging behaviour: blue—regular swimming outside the wake, red—altered gait in hydrofoil wake, green—swimming regimes which can not be uniquely identified. In the absence of the vortex street, hence with zero blade oscillation frequency, fish only swim regularly.

The proportion of the time spent in different swimming modes, obtained by analysis of fish swimming videos, is shown in Fig. 2 for the three individuals that exhibited an altered gait.

**Figure 3:**
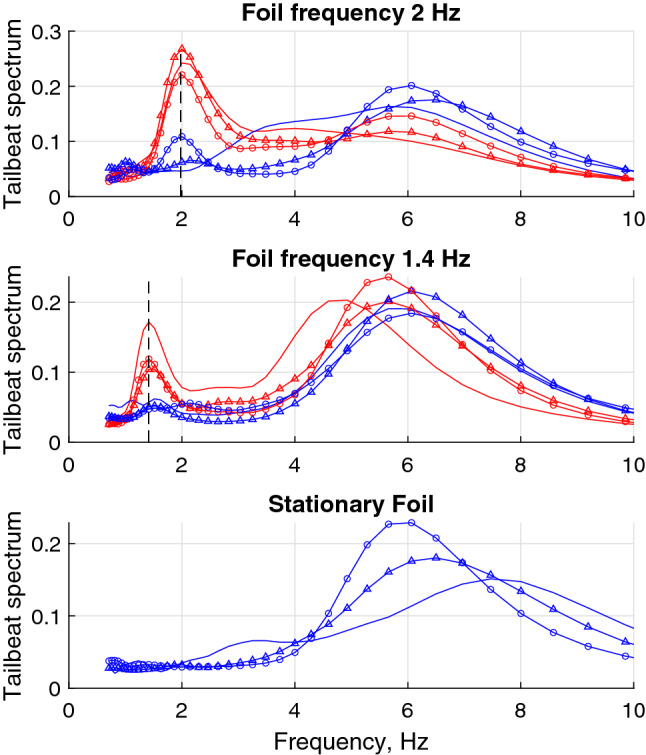
Frequency spectra of the curvature of fish body in different swimming regimes. Blue—regular swimming outside the wake, red—altered gait in the hydrofoil wake. Each marker corresponds to an individual fish. The vertical dashed line indicates foil oscillation frequencies. A clear peak at the hydrofoil oscillation frequency is evident in the altered gait cases, corresponding to the fish tuning their body shapes to the vortices.

To quantitatively examine the two swimming gaits (swinging and regular), a wavelet analysis of the curvature of fish muscle was performed. After studying dissections of the sub-carangiform swimmers, the fish muscle has been assigned to span from 1/3 to 13/16 of the body length, measured from the front of the head. The power spectrum of the tailbeat amplitude in the different regimes is shown in Fig. 3 in arbitrary units. It can be observed that, consistent with Fig. 1, the tailbeat frequency in regular swimming, with values typically between 5 and 6 Hz, is notably higher than the frequency of body bending tuned to that of the passing vortices, which is close to the frequency of the hydrofoil oscillation.

To quantitatively assess the difference in energy expenditure between regimes, we analysed the curvature of the fish centerline and estimated the fish propulsive power to be proportional to the rate of the volumetric contraction of the muscles. Further details of the method of propulsive power estimation are presented in the supplementary material.

**Figure 4:**
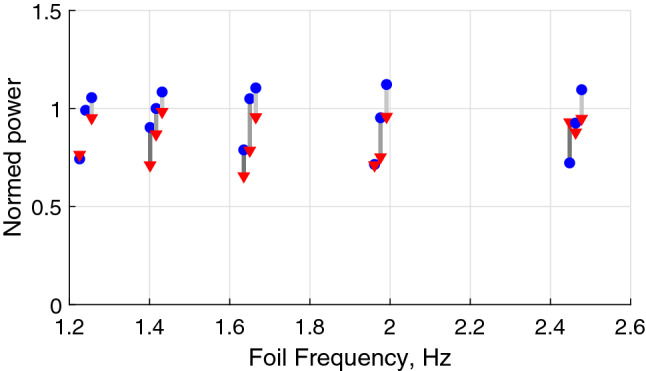
Power expenditure by fish in different swimming modes divided by that in the absence of the vortex street: blue circles—regular swimming outside the wake, red triangles—altered gait in hydrofoil wake. Pairs corresponding to the same fish are joined by grey lines and shifted horizontally to improve clarity. A consistent reduction of the energy expenditure can be observed for all three fish at the foil frequencies of 1.4 Hz, 1.6 Hz and 2.0 Hz.

It has been found that, in the foil frequency range between 1.4 and 2 Hz, the estimated energy expenditure in the swinging regime is consistently lower than that when swimming regularly outside of the wake, with an average benefit reaching 25% as shown in Fig. 4. In the current arrangement, in order to use vortices, the fish moves to the inner region of the wake where it experiences up to 20% higher flow velocities (and hence 30–40% higher drag), as illustrated by velocity profiles in Fig. 1. Therefore, the power benefit of swinging at vortices in thrust-neutral Kármán street may reach 50%.

## Discussion

**Figure 5:**
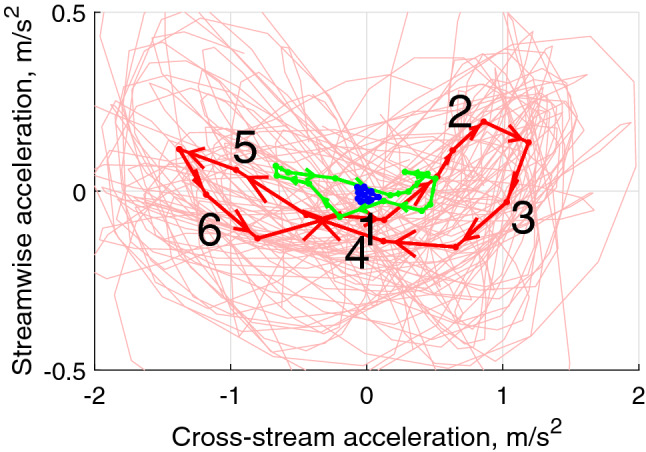
Phase-averaged trajectory of an individual fish in the space of cross-stream/streamwise accelerations in different swimming modes: blue—regular swimming outside the wake in the uniform flow field, red—swinging in the wake, green—swimming regimes which can not be uniquely identified. The non-averaged trajectory in the swinging regime is shown by the pale red line. Observe the well-defined figure of eight in swinging mode, when the fish ’pushes off’ the cross-stream flow to produce positive streamwise acceleration. The figure shows phase trajectory of fish #1 recorded for 22 s. The foil oscillation frequency is 2 Hz.

**Figure 6:**
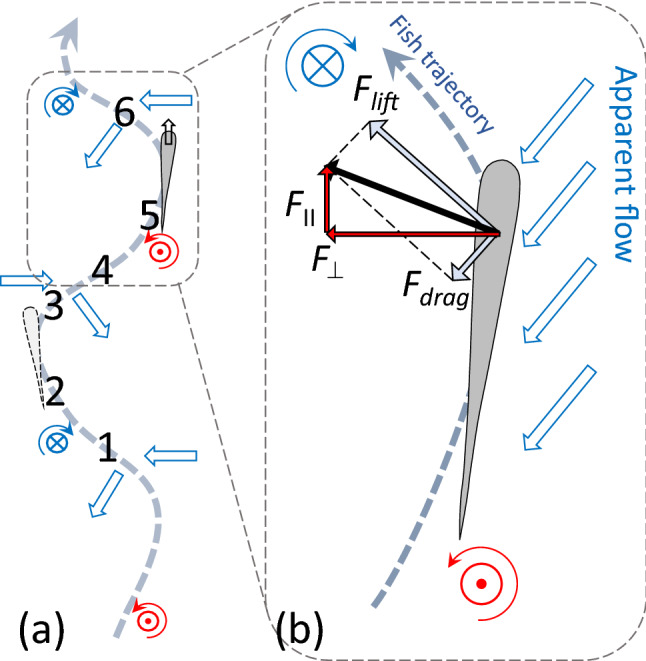
Forces acting on the fish in the flow composed of the uniform component and the component added by vortices. The vector sum of lift and drag forces has a positive streamwise component assisting fish propulsion. The cross-stream component of the hydrodynamic force leads to fish following an undulatory trajectory with the acceleration shown in Fig. 5.

To provide insight into the physics of the altered swimming gait, the accelerations of the fish centre of volume are examined. Figure 5 demonstrates the phase trajectories obtained by plotting the streamwise acceleration against the cross-stream acceleration. The streamwise position of the fish with respect to the vortices was used to combine parts of the trajectory for averaging. It is visually clear that in the swinging regime, the streamwise acceleration correlates well with the cross-stream acceleration and both acceleration components are an order of magnitude larger than with the regular gait. The range of accelerations in both the streamwise and cross-stream directions also differ significantly between the regular regime (blue) and the altered gait (red line), with a barely noticeable range of accelerations in the regular swimming case. We postulate that the fish utilize the periodic cross-stream flow generated by the vortices to enhance their thrust force and hence accelerate in the streamwise direction, reducing their total energy expenditure. This leads to the positive loops of the streamwise acceleration at the cross-stream acceleration maxima (2–3 and 5–6) shown in Fig. 5 with the relative spatial fish-vortex position shown in Fig. 6a. At position 4, where the fish does not experience the cross-flow, the cross-stream acceleration is shown to be zero and consequently the streamwise acceleration is negative. As the accelerations must form a closed loop for the position to be maintained in a time averaged sense, a characteristic figure-eight shaped trajectory is formed.

By examining the projections of the hydrodynamic forces acting on the fish, the explanation of the mechanism can be expanded. As shown in Fig. 6a, the cross-stream component of the flow causes the fish to swing from left to right. At points marked 2 and 5, the fish body is exposed to flow with a non-zero cross-stream component, resulting in an increase in both the lift and drag forces which, for an appropriate angle of attack, produces a net forward force as shown in Fig. 6b. For reference, the position of points 1–6 marked in Fig. 5 are shown in Fig. 6a. Here, points 1 and 4 correspond to the fish position in the inner region of the wake, where the body is not extracting energy from the lateral flow and hence decelerating in the streamwise direction.

**Figure 7:**
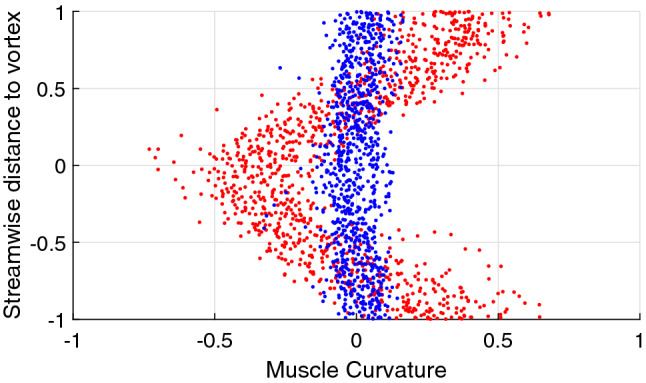
Fish muscle curvature at different phases of the fish streamwise position with respect to vortices. Blue—regular swimming outside the wake, red—swinging in the wake. Clear dependence of the curvature on the position in the vortex street suggests fish adjust the body shape to improve efficiency of interaction with the cross-flow. The figure shows data acquired from fish #1 swinging in the hydrofoil wake with an oscillation frequency of 2 Hz.

In addition to adjusting their cross-stream position with respect to the vortices to reduce energy expenditure, the curvature of the fish body has also been observed to adapt to the vortical flow field. Figure 7 illustrates an example of the normalised streamwise position to vortex versus the amplitude of the curvature of the muscular body. In the swinging regime, the body curvature can be seen to have a maximum absolute value when the fish head approaches the vortex. This also corresponds to the instance of maximum cross-stream acceleration, suggesting that the fish adjusts its curvature to increase its lift when exposed to lateral flow and hence the force component parallel to the freestream flow direction, enhancing the thrust gained from the interaction with the vortex. The adjustment of the body curvature may also serve to stabilise the additional yawing moment associated with the increased hydrodynamic forces.

**Figure 8:**
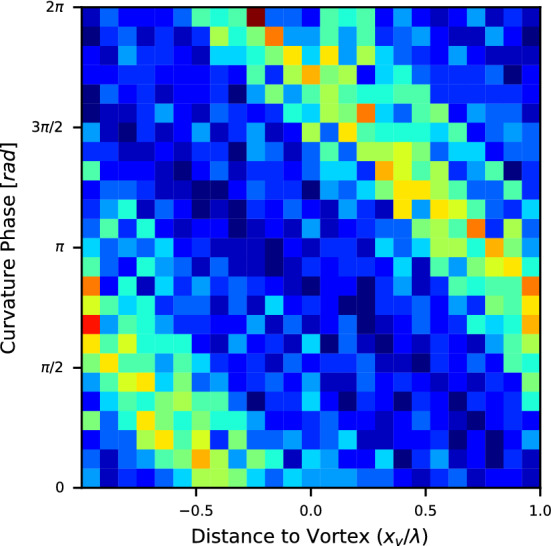
Histogram of extracted fish muscle curvature phase versus non-dimensional distance to vortex. $\lambda$ denotes the distance between vortices, while the distance of the fish centre of volume to the next upstream vortex is $x_{v}$. The extracted phase can been seen to vary close to linearly with distance to vortex. The figure shows data acquired from fish #1 swinging with a foil oscillation frequency of 2 Hz.

The phase of the additional oscillatory component of the body curvature developed at the hydrofoil oscillation frequency can be extracted by band-pass filtering the body curvature amplitude in the vicinity of the foil oscillation frequency, followed by computing the Hilbert transform of the filtered signal. Fig. 8 demonstrates an example of the extracted curvature phase versus the distance to the vortex for the swinging regime. Similar to the observations of Li et al.^29^, the curvature phase can be seen to vary linearly with distance to vortex.

Evaluating the propulsive force is difficult not only as calculating the required lift and drag coefficients is challenging with a highly 3-dimensional flow, but also because the Strouhal number based on the fish length varies in the range 0.4–0.8, which suggests that time-dependent effects need to be accounted for. However, to illustrate the feasibility of the cross-flow-based propulsion mechanism, we evaluate the characteristic time $\tau$ for the fish to reverse the cross-stream velocity in the cross-stream flow opposing its motion, i.e. the time corresponding to fish dynamics between points 1 and 4 or 4 and 6 in Fig. 6a. We write the Newton’s second law as

1 $$\begin{aligned}&F_\perp \sim m_{{\rm fish}}\frac{U_\perp }{\tau }, \end{aligned}$$

2 $$\begin{aligned}&A_{{\rm side}}C_{\rm d}\,\rho _{{\rm water}}\frac{U_\perp ^2}{2} \sim \rho _{\rm{fish}} V_{{\rm fish}}\frac{U_\perp }{\tau }, \end{aligned}$$

where $F_\perp$ is the cross-flow hydrodynamic force acting on the fish, $U_\perp$ is the cross-flow velocity of the fish, and $\tau$ is the characteristic time of the fish cross-flow acceleration/deceleration. Assuming fish density similar to that of water, drag coefficient $C_d$ being similar to that of a cylinder at 1, fish width $W_{\rm{fish}}\sim 1\,\hbox {cm}$, and the cross-flow velocity $U_\perp \sim 0.1\,\hbox {m}/\hbox {s}$ measured by tracking, we have

3 $$\begin{aligned} \tau \sim \frac{2 W_{{\rm fish}}}{C_{\rm d} U_\perp }\sim 0.2\,\text {s,} \end{aligned}$$

which is consistent with under half of a cycle spent in phases of high lateral acceleration (segments 2–3 and 5–6 in Figs. 5 and  6a) when the frequency of vortices passing the fish is 3–4 Hz (foil flapping frequency 1.6–2 Hz respectively) at which fish consistently swings in the wake. When the time for the fish to switch the sign of the cross-flow velocity is close to the time between vortices of the opposite sign, extracting the energy from the alternating cross-flow and converting it to propulsion proves efficient. This estimated frequency range agrees with the peak in the proportion of times fish displayed the swinging gait seen in Fig. 2 and with the most pronounced energy benefit of swinging compared to the regular gait seen in Fig. 4. The scaling for location of the optimum in the vortex frequency (Eq. 3) agrees with experiments reported in Akanyeti and Liao^18^, where a similar Kármán gaiting frequency was observed with three times larger fish in a flow of three times the velocity.

In conclusion, rainbow trout have been observed to swim with an altered gait in a thrust generating reverse von Kármán vortex street produced by an oscillating hydrofoil, which can be associated with the flow generated by a large leader fish. The rainbow trout synchronise their body movements with the vortices and exhibit a large cross-flow oscillation in their centre of mass position. This leads to a reduction in energy expenditure, exceeding the loss incurred due to the higher streamwise velocity in the wake. The altered gait differs from the behaviour of similar-sized fish swimming in schools as it occurs at frequencies significantly lower than the natural tailbeat frequency of a fish swimming alone or in a school. Rather than slightly tuning the tailbeat frequency to match that of a leader, fish use their body inertia and orientation to exploit the non-equilibrium hydrodynamic forces generated when exposed to the alternating cross-flow. This allows them to generate additional thrust and reduce their energy expenditure. Despite visual similarity, the swinging is different from the extensively studied pitching or heaving^30–32^ in the sense that both lateral and angular positions are achieved by smart orientation of the whole fish with respect to the surrounding flow structures rather than by application of an external force to a propulsive fin. The shape of the fish body in different gaiting phases maximises the propulsive component of force and provides steering to compensate imbalances in the yawing moment.

The mechanics of fish extracting energy from the alternating cross-flow is similar to that of dynamic soaring of albatross, where the stationary gradient of the cross-flow in the atmospheric boundary layer is exploited. The difference is that albatross actively change height moving between fast and slow cross-flow, while fish follow the upstream path while vortices pass by.

## Methods

*Juvenile rainbow trout* (*Oncorhynchus mykiss*, $\hbox {n}=34$, total length of $55\pm 3\ \hbox {mm}$), sourced from the Bibury Trout Farm (UK), were maintained within the Cardiff University Aquarium at $14\pm 1^{\circ }\hbox {C}$. Motion tracking experiments were performed in a 0.3 m wide, 0.5 m long and 0.27 m deep test section of the recirculating flume at the hydraulic laboratory, Cardiff University. An oscillating hydrofoil constructed with a NACA 0012 section and a chord length of 75 mm was used to generate a series of vortices in the test section. The vertical span of the hydrofoil was 200 mm, while the water depth was maintained at 230 mm. The mean flow velocity was maintained at 0.14 m/s. To maintain the trout within the field of view of the camera, the upstream end of the test section was bound by a honeycomb flow straightener of 5 mm cell size and a plastic mesh at the downstream end. During each test, a single trout was observed for 5 min interacting with the vortex street after a 5 min acclimatisation period.

### Ethics declaration

Fish behavioural experiments were approved by Cardiff University Animal Ethics Committee and conducted and reported following the ARRIVE guidelines under Home Office License PPL 303424.

*Images* of the fish were captured from above the flume with a FLIR Grasshopper camera at the resolution of $2048\times 2048$ pixels and the frame rate of 45 Hz. The images were processed to allow the extraction of the fish centreline.

*To characterise the flow field* generated by the oscillating hydrofoil, ultrasonic velocity measurements and flow visualisation was performed in the absence of fish. A MET-FLOW 8 MHz Ultrasonic Velocimetry Profiler (UVP) probe was traversed across the test section in 2 mm increments. Velocity measurements were obtained with a sampling frequency of 200 Hz at 10 positions along the UVP beam, which was inclined at $24^{\circ }$ to the freestream flow direction.

*Flow visualisation* of the vortex street was performed by manually injecting food colouring at the leading edge of the hydrofoil close to the mid-depth position. Images were captured with a FLIR Grasshopper camera mounted above the flume, as for the capture of the fish kinematics.

*The energy expenditure* of the fish was linked to the contraction of muscles along the body associated with bending in horizontal direction. It has been assumed that the work performed by muscles is defined by the textbook value $E=F\cdot \Delta L$ and that force applied by the muscle per unit cross-sectional area is constant. When the muscle cross-section and contraction varies, the power exhibited by the muscle is defined by the integral of the absolute value of the volume change associated with muscle contraction along the fish length. To calculate the instantaneous power, the rate of change of the curvature $\kappa$ of centreline from 1/3 to 13/16 of the body length *L* was multiplied by the moment of area *A* of the muscle calculated from fish cross-sections and then integrated along the length of the muscle as follows:

4 $$\begin{aligned} W\propto \int _{\frac{1}{3}L}^{\frac{13}{16}L} \left| \frac{d\kappa }{dt}\right| \left( \iint _{A(\ell )}|x|\,dx\,dy\right) d\ell , \end{aligned}$$

where $\ell$ is the coordinate along the fish, *x* is directed horizontally perpendicular to the fish plane of symmetry, and *y* is vertical. The contours of fish cross-section are evaluated from a dissected species. The power has been evaluated in arbitrary units and not converted to Watt. While the location of the muscle along the fish body between 1/3 and 3/16 is somewhat arbitrary, the power expenditure does not significantly depend on these numbers because the energy produced close to the ends of the interval vanishes: The body curvature is nearly zero from the head to the vicinity of 1/3 and the muscle cross-section is nearly zero from the vicinity of 3/16 to the tip of the tail. Due to the lack of literature on the calculation of the energy expenditure from the fish body shape, we have attempted a variety of methods which all lead to the same qualitative result: In the swinging regime, fish spends notably less energy.

## Supplementary Information

Supplementary Information.

Supplementary Video 1.

Supplementary Video 2.

Supplementary Video 3.

## Data Availability

The datasets used and analysed during the current study available from the corresponding author on reasonable request.
