# Supplementary material for: An inertial mechanism behind dynamic station holding by fish swinging in a vortex street
Source: Sci Rep. 2022 Jul 25;12:12660. doi: 10.1038/s41598-022-16181-8 (PMC9314336; doi:10.1038/s41598-022-16181-8)
Supplement: Supplementary file 1 — Supplementary Information. [file 41598_2022_16181_MOESM1_ESM.pdf]

SUPPLEMENTARY MATERIAL for the article

An inertial mechanism behind dynamic station holding by fish swinging in a vortex street

S. Tucker Harvey, V. Muhawenimana, S. Muller, C. A. M. E. Wilson and P. Denissenko

June 30, 2022

Generation of Vortex Street

The study on swimming kinematics of rainbow trout in the wake of a pitching hydrofoil was conducted in the hydraulic laboratory at Cardiff University between 9am and 5pm in August 2019. To characterise the flow, an Ultrasonic Velocity Profiler (UVP) and flow visualisation were utilised. The experimental setup comprised of a 10 m long and 0.3 m wide recirculating flume (Fig. S1). The flow depth was kept at 0.23 m and the cross-sectional averaged velocity U_0 was set at $0.14\,\mathrm{m\,s^{-1}}$. An oscillating hydrofoil constructed with a NACA 0012 cross section of the chord length of 75 mm and the vertical span of 200 mm, positioned 3.8 m downstream of the flume inlet was used to generate the inverse Von Kármán wake. The pitching motion of the hydrofoil was actuated using a NEMA 24 stepper motor, which was connected to the axle of the hydrofoil located at 1/10 chord length from the leading edge via a four bar linkage. The oscillation frequency was varied from 1.25 Hz to 2.5 Hz. The flow field was investigated with no fish present and then synchronised with fish experiments using movement of the foil. Ultrasonic Velocimetry Profiler (UVP) measurements comprised of 101 profiles across the width of the flume spaced 2mm laterally. Profiles of the mean streamwise velocity are shown in Fig. S2. The near wake was visualised using Fluorescent Fwt red and Flt yellow/green (Cole-Parmer Instrument Ltd.), which was injected at the leading edge at height of the vertical centre of the oscillating hydrofoil.

Fish Treatment and Tracking

Juvenile rainbow trout (*Oncorhynchus mykiss*, n=34, full length 55±3 mm), sourced from Bibury Trout Farm (UK), were maintained within the Cardiff University Aquarium at 14±1°C. The test section, shown in Figure Fig. S1, was 0.5 m long, reaching 0.6 m downstream of the hydrofoil trailing edge. The upstream end of the test section was bounded by a honeycomb flow straightener of 6 mm cell size and a plastic mesh bounded the downstream end. Fish swimming kinematic images were recorded with a FLIR Grasshopper camera mounted above the flume, capturing images with a resolution of 2048×2048 pixels at a frame rate of 45 Hz. A window made of transparent acrylic was mounted at the top to eliminate image distortion by waves at the free surface. The window also housed white LED strips used to illuminate the test section. Two additional LED spotlights were placed on either side of the flume. Prior to the experiment, the water was dechlorinated using Seachem Prime Concentrated Conditioner and chilled to 14°C. Each fish was transferred to the flume and given a 10 min acclimatisation period under the test flow conditions, with the hydrofoil stationary. Each trial lasted 30 min in which the fish was exposed to 6 types of vortex street generated by hydrofoil in random order, lasting 5 min each. Fish behavioural experiments were approved by Cardiff University Animal Ethics Committee and conducted following the ARRIVE guidelines under Home Office License PPL 303424.

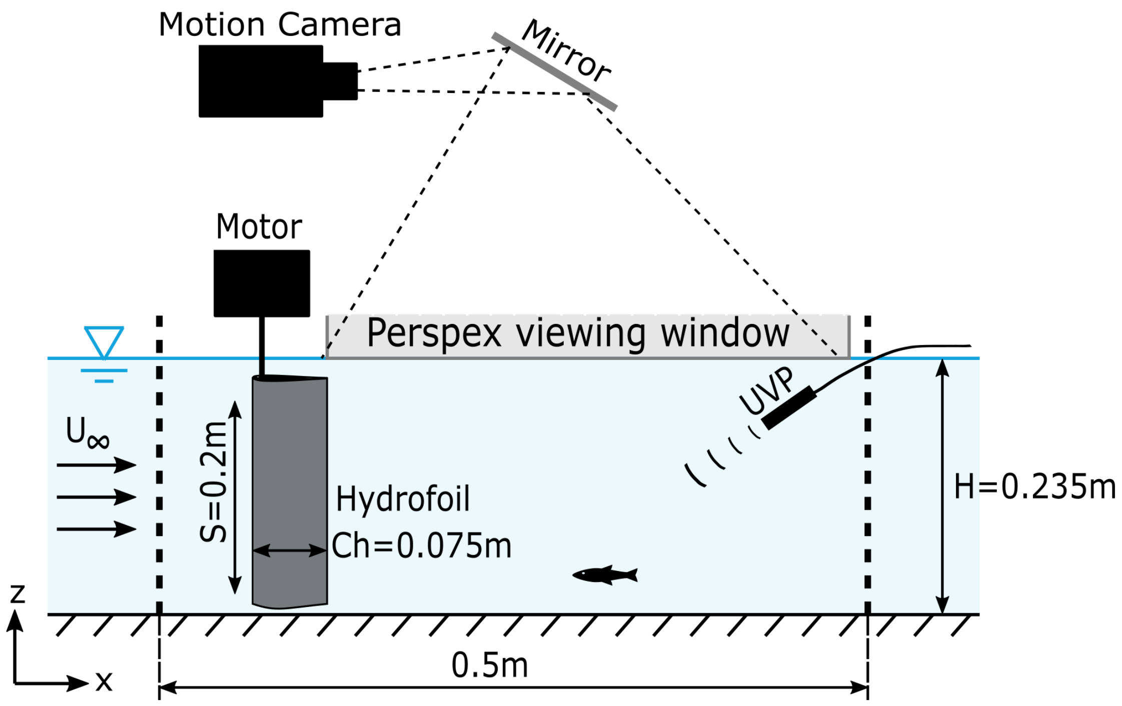

Figure S1: Experimental setup, side view: flume, oscillating foil, and a rainbow trout.

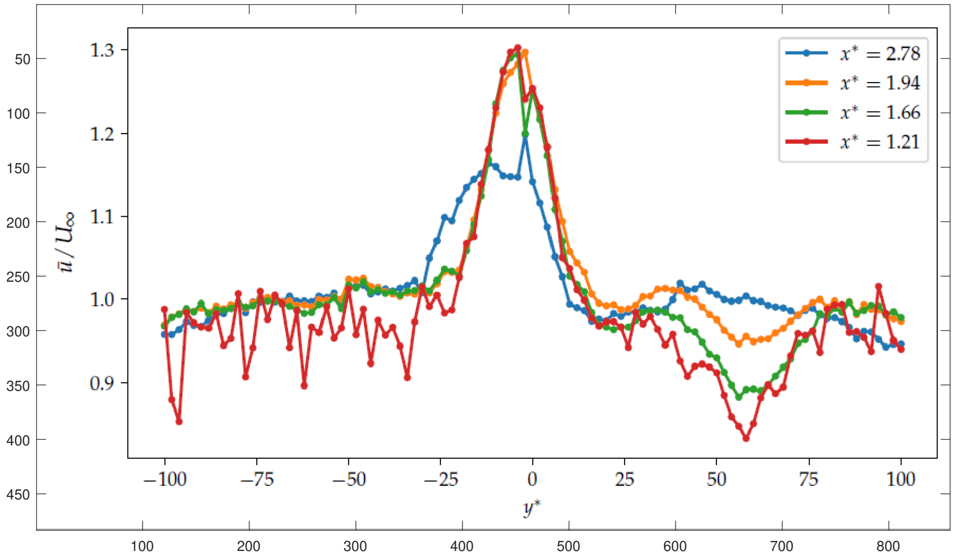

Figure S2: Profiles of the mean flow velocity measured by UVP. Observe up to 30% increase in the central area associated with the thrust-generating Kármán vortex street.

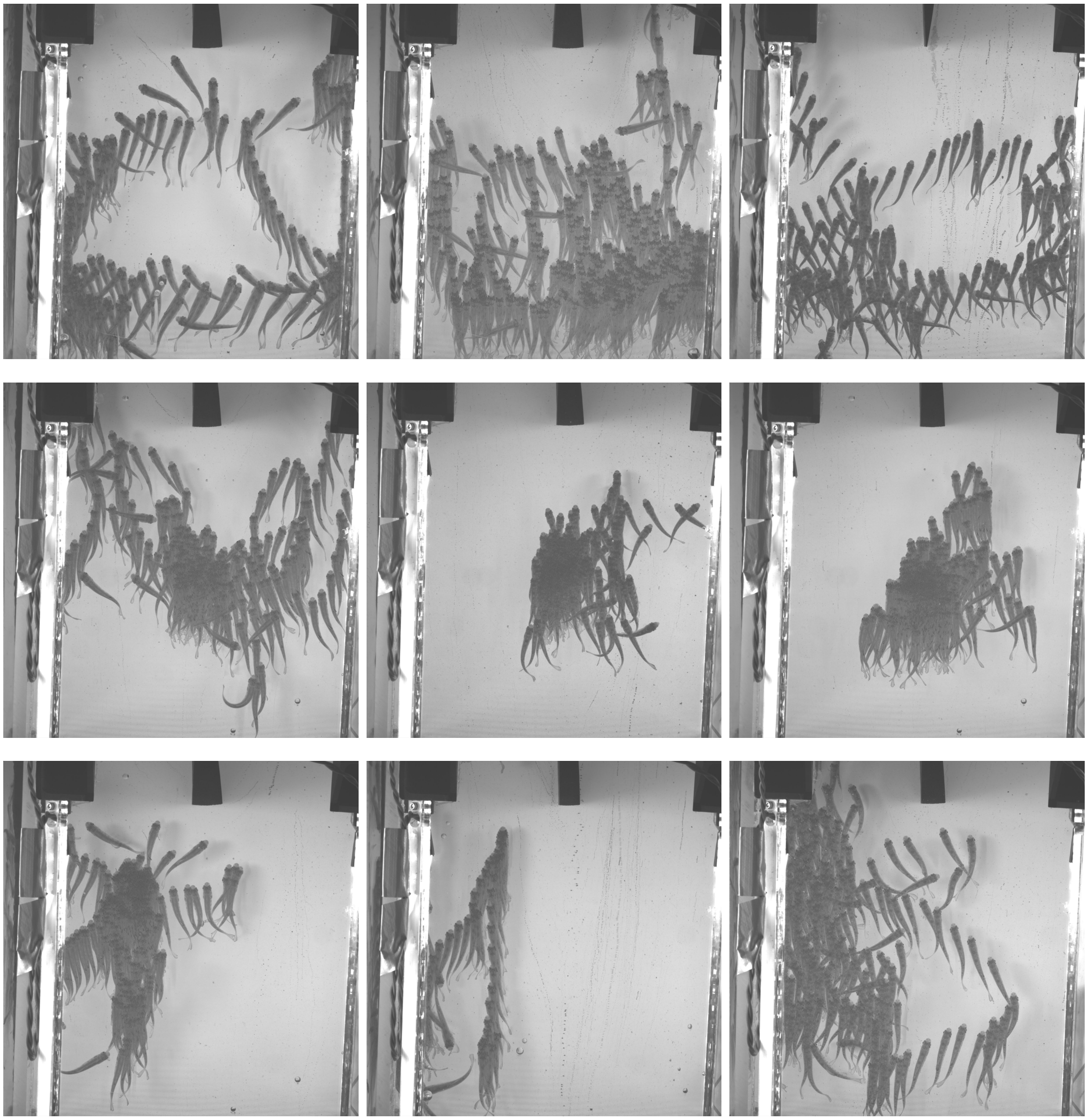

Figure S3: Superposition of a single fish positions acquired 3 times a second to illustrate probing the whole field (top row), mostly swinging in the vortex street (middle row), and avoiding the vortex street (bottom row). The multiple hydrofoil positions upstream correspond to its oscillations.

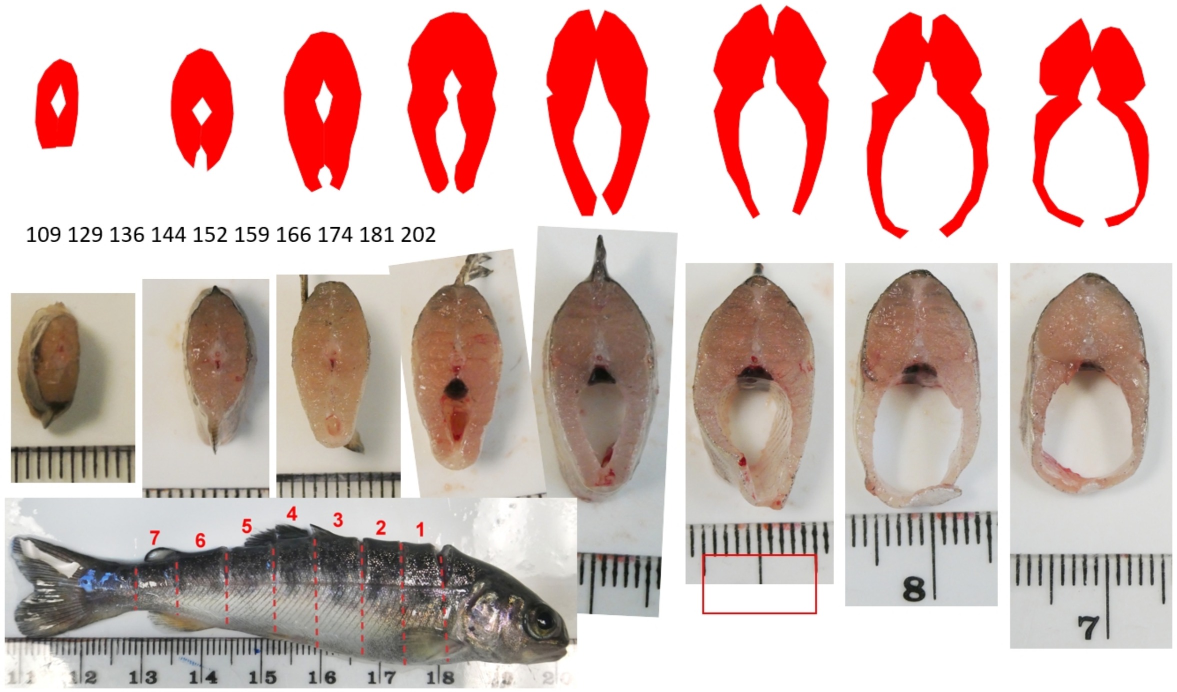

Figure S4: A trout has been dissected to quantify the muscle cross-section and calculate moments of the area.

Calculation of energy expenditure

To calculate fish energy expenditure, it has been assumed that the work performed by muscles is defined by the textbook value $E = F \cdot \Delta L$ and that force applied by the muscle per unit cross-sectional area is constant. When the muscle cross-section and contraction varies, the power exhibited by the muscle is defined by the integral of the absolute value of the volume change associated with muscle contraction along the fish length. To calculate the instantaneous power, the rate of change of the curvature κ of centreline from 1/3 to 13/16 of the body length was multiplied by the moment of area of the muscle calculated from fish cross-sections and then integrated along the length of the muscle. The power has been evaluated in arbitrary units and not converted to Watt.

$$W \propto \int_{\frac{1}{3}L}^{\frac{13}{16}L} \left| \frac{d\kappa}{dt} \right| \left(\iint_{A(\ell)} |x| \; dx \; dy \right) d\ell, \tag{1}$$

where ℓ is directed along the fish, x is the directed horizontally perpendicular to the fish plane of symmetry, and y is vertical. The contours of fish cross-section are evaluated from a dissected specie as shown in Fig. S4. While the location of the muscle along the fish body between 1/3 and 3/16 is somewhat arbitrary, the power expenditure does not significantly depend on these numbers because the energy produced close to the ends of the interval vanishes: The body curvature is nearly zero from the head to the vicinity of 1/3 and the muscle cross-section is nearly zero from the vicinity of 3/16 to the tip of the tail. With the lack of papers on calculation of the energy expenditure from the fish body shape, we have attempted a variety of methods which all lead to the same qualitative result: In the swinging regime, fish spends notably less energy.

Plots for the three selected fish at all hydrofoil frequencies

For the watchful reader to reflect on the fish swimming regimes we observe and to make own conclusions, plots similar to those in the main body, and several more, are presented for all three of selected fishes at all 6 frequencies of hydrofoil oscillation.

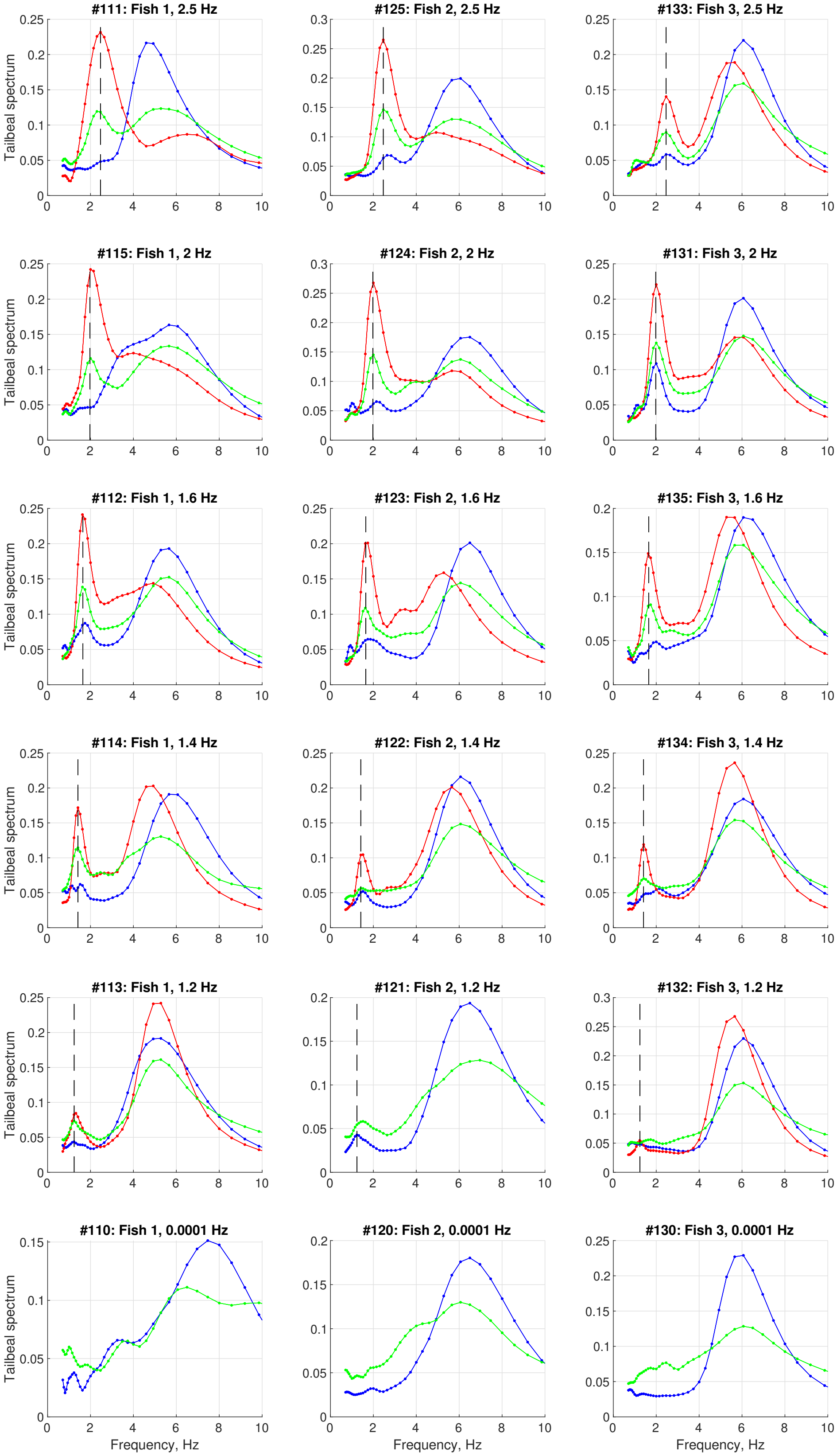

Figure S5: Averaged power spectra of the tailbeat motion. Blue - Regular swimming, Red - Swinging, Green - Other parts of the recording.

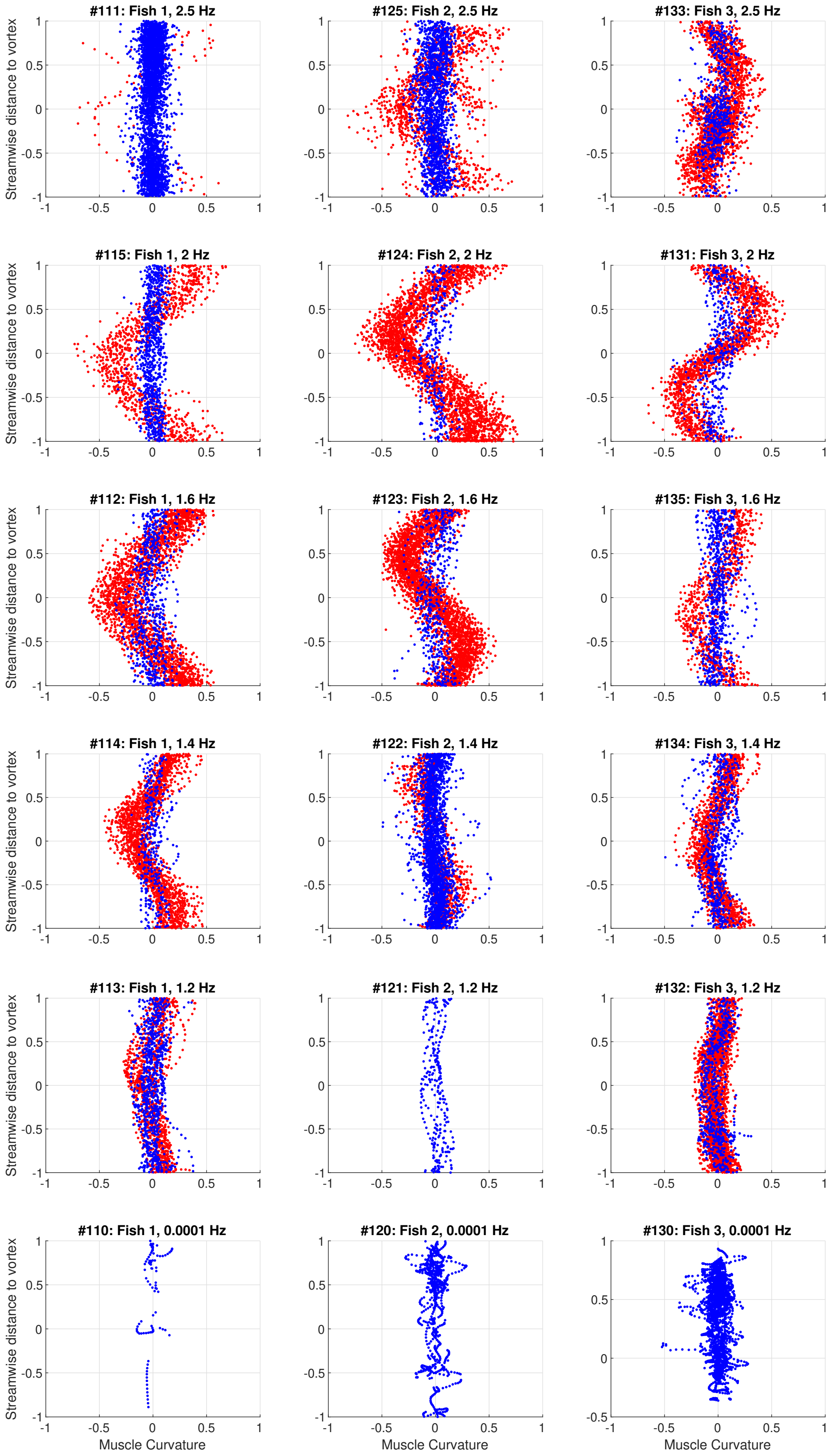

Figure S6: Muscle curvature vs. streamwise position of the fish head with respect to vortices: Blue - regular swimming, Red - swinging.

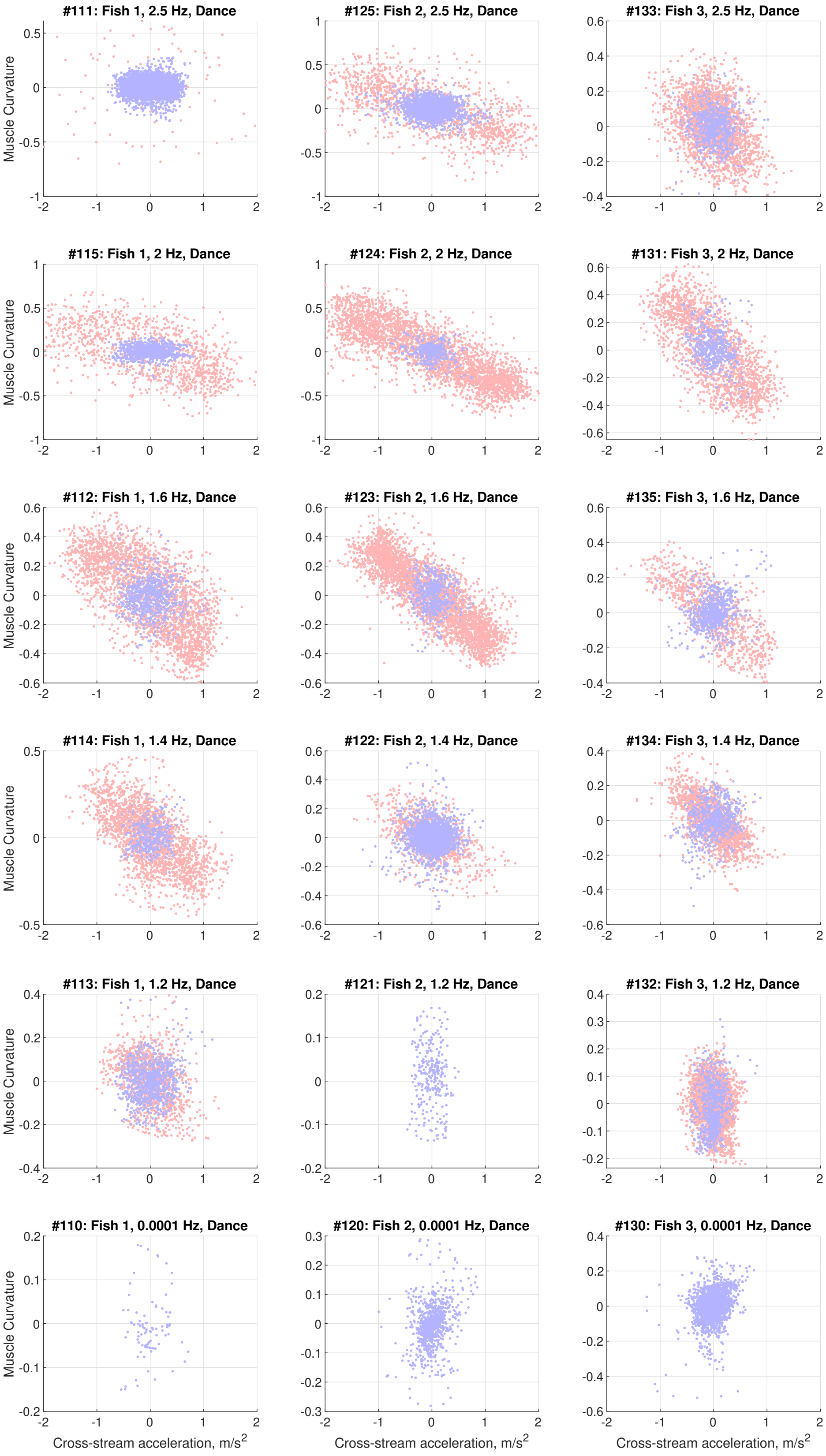

Figure S7: Correlation between fish muscle curvature and the cross-flow acceleration: Blue - regular swimming, Red - swinging.

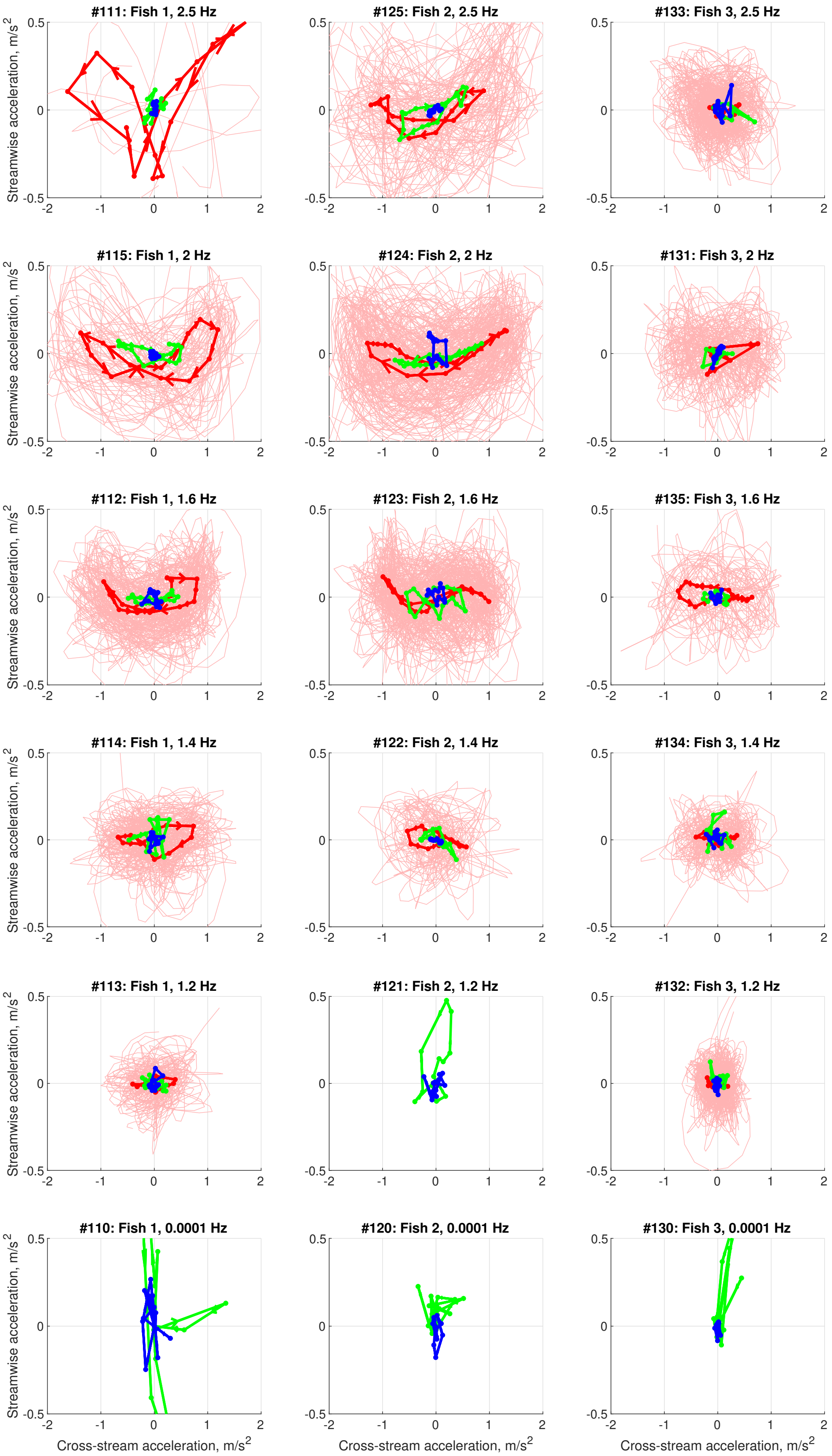

Figure S8: Phase-averaged trajectories of fish in the space of cross-stream and stream-wise acceleration. Blue - Regular swimming, Red - Swinging, Green - Other parts of the recording. With data being noisy, not all cases where swinging behaviour is visually obvious produce clear phase trajectories when averaged.

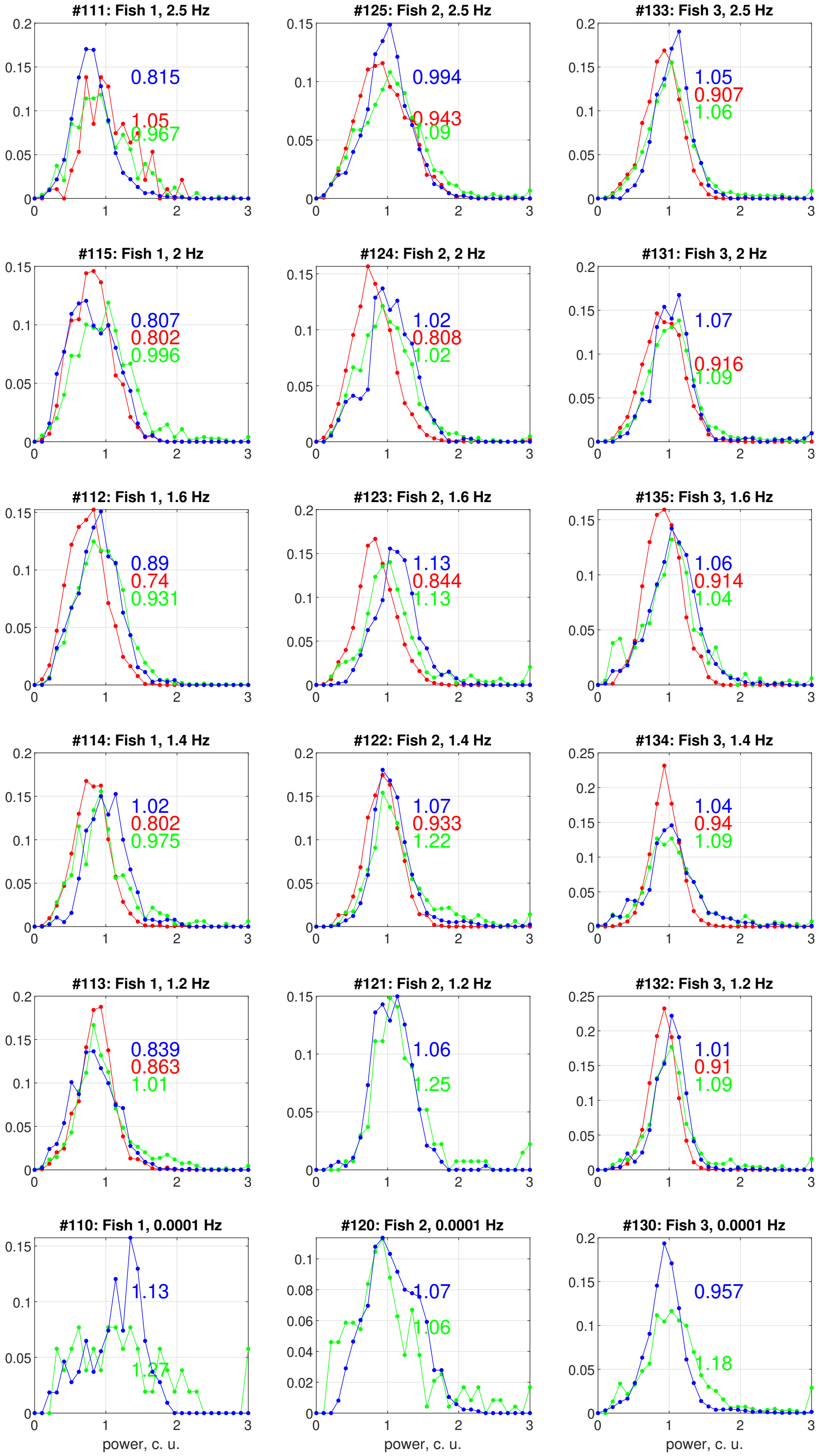

Figure S9: Histogram of fish instantaneous power calculated from the rate of change of the centerline curvature: Blue - regular swimming, Red - swinging, Green - Other parts of the recording.

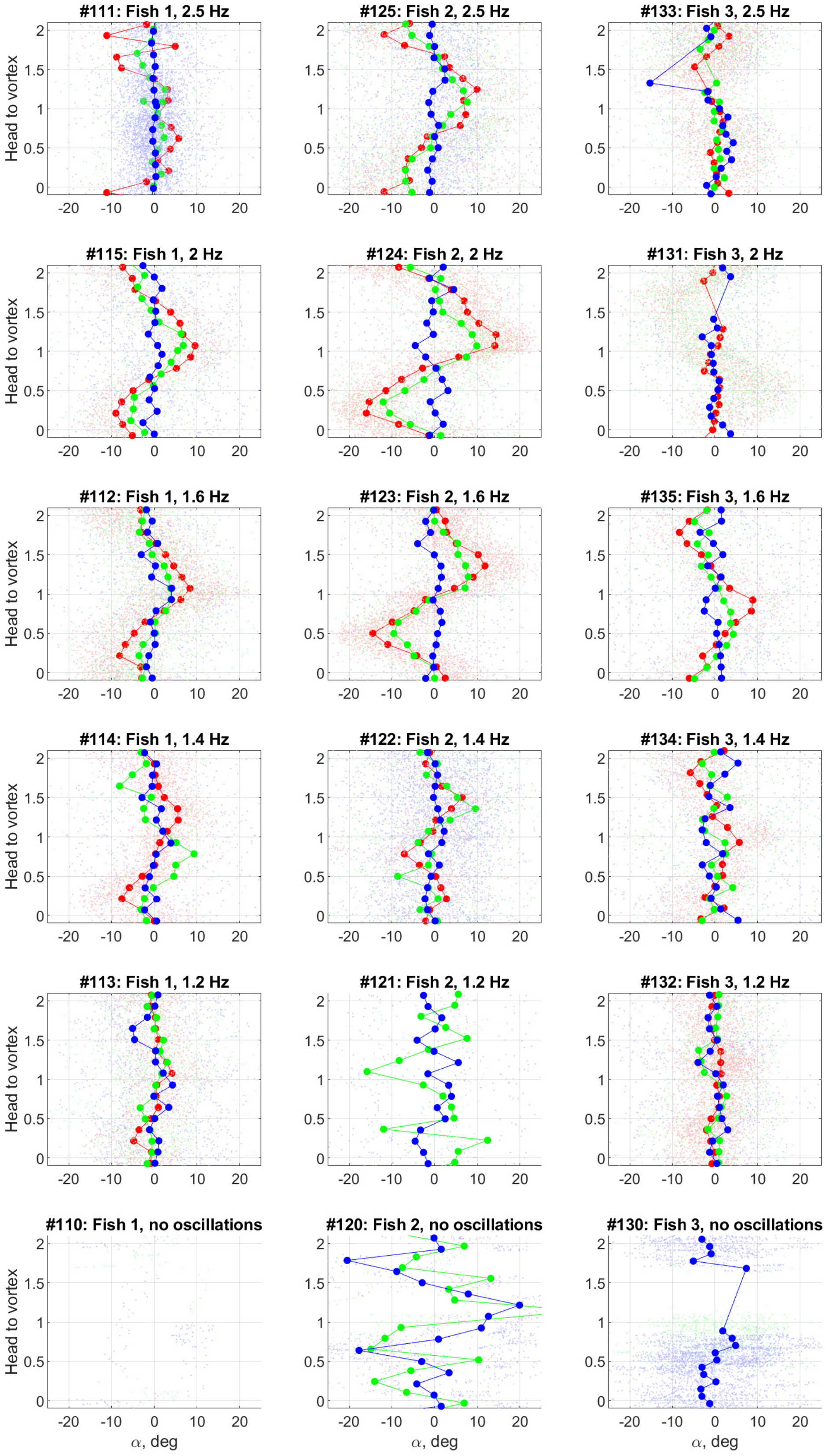

Figure S10: Angle of fish body to the streamline direction

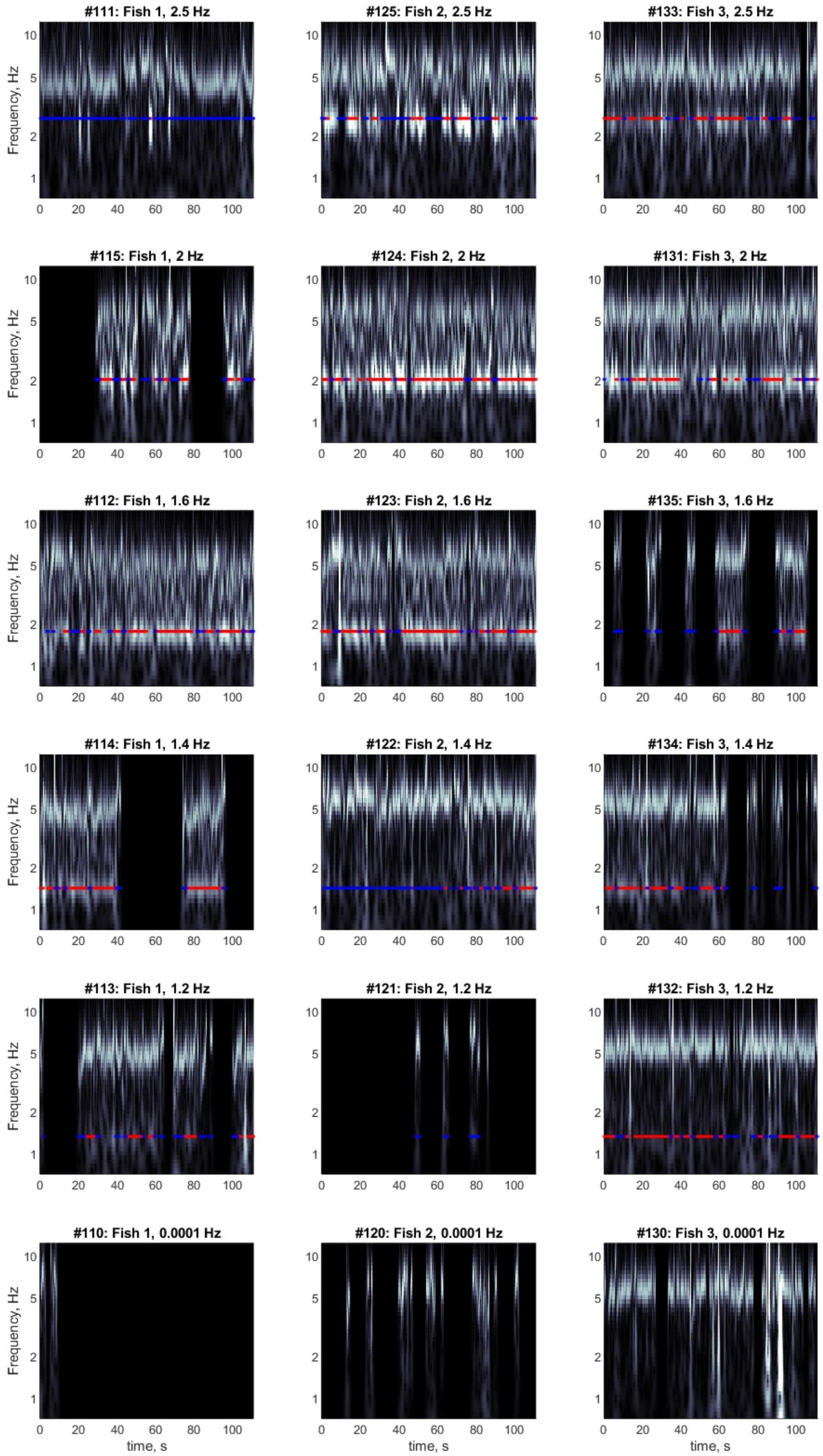

Figure S11: Wavelet analysis of the fish body curvature: Time dependence of the power spectra. Markers are plotted at the foil oscillation frequency. Red markers correspond to the ”swinging” gait, blue markers to other gaits. Observe presence of the foil frequency (light areas) in swinging gait and absence of the foil frequency (dark gaps at the corresponding frequency range) in other gaits.
